# Hyperthermic Intraperitoneal Chemotherapy Following Cytoreductive Surgery for Colorectal Peritoneal Carcinomatosis Patients: A Review

**DOI:** 10.7759/cureus.32440

**Published:** 2022-12-12

**Authors:** Sarah S Alhumaidan, Abeer M Alharbi, Ayesha Farhana Syeda, Fatimah A Alghaidani, Manal M Almutairi, Nourah A Alharbi, Reham K Alenezi

**Affiliations:** 1 Faculty of Pharmacy, Unaizah College of Pharmacy, Qassim University, Unaizah, SAU

**Keywords:** colorectal peritoneal carcinomatosis, colorectal cancer, cytoreductive surgery, hipec, hyperthermic intraperitoneal chemotherapy

## Abstract

Colorectal peritoneal carcinomatosis (CPC) is an advanced malignancy and is typically associated with a poor prognosis. Hyperthermic intraperitoneal chemotherapy (HIPEC) following complete cytoreductive surgery (CRS) is a novel, advanced loco-regional treatment for colorectal cancer that is currently being used to treat peritoneal carcinomatosis (PC). The present review aims to describe the evidence-based literature on the efficacy and safety of this treatment approach in patients with PC originating from colorectal cancer and to summarize its complications. All published literature regarding the efficacy of HIPEC for the treatment of CPC was reviewed; 16 studies were included in this paper. The overall survival rate for the HIPEC group ranged from 63% to 93%. The overall median survival for the HIPEC and non-HIPEC groups ranged from 13 to 60.1 months and 12.6 to 41.2 months, respectively. The overall median survival of patients in the HIPEC group was comparatively better than those in the non-HIPEC group. There was insufficient evidence to suggest whether this treatment regimen was associated with a high or low morbidity rate in comparison to other groups. However, the mortality rate associated with this treatment regimen was low. In conclusion, the present data provide insufficient evidence regarding the beneficial effects of using HIPEC following CRS treatment. Therefore, further studies are required to determine the benefits of HIPEC for CPC patients.

## Introduction and background

Colorectal cancer (CRC) is one of the most commonly diagnosed cancers worldwide, with 1.93 million new cases and 916,000 deaths worldwide in 2020 [1]. Colorectal peritoneal carcinomatosis (CPC) is a peritoneal surface malignancy that can be secondarily induced by the spread of cancer cells from pre-existing cancer [2]. CPC from CRC is a manifestation of metastatic cancer [3]. One study found that, in 12 patient reports, the prevalence of peritoneal metastasis during potentially curative surgery for primary CRC varied between 3% and 28%. This variation could be due to the use of different cancer cell detection techniques. PC was detected in 7% of patients during resection surgery, in 4% to 19% during later follow-ups, in 44% during second laparotomies undertaken for CRC recurrence, and in 40% to 80% of patients who succumb to CRC [4].

As CPC is diagnosed more often, it necessitates the development of new research to discover new and more effective modalities of treatment, as systemic chemotherapy is known to have poor efficacy in PC treatment. Traditionally, treatment involves surgical tumor excision followed by systemic chemotherapeutic and/or radiotherapeutic agents. However, the overall survival rate is relatively poor due to peritoneal surface involvement and the insufficient amount of systemic chemotherapy that reaches the peritoneal cavity [5].

CPC is an advanced malignancy typically associated with a poor prognosis. Malignant tumors on the peritoneal surface are the main cause of morbidity and mortality and are, therefore, the primary concern during cancer treatment. Although PC is classified as a metastatic disease, it represents a special disease pattern and is considered a localized disease confined to the abdominal cavity. Complete cytoreductive surgery (CRS) and hyperthermic intraperitoneal chemotherapy (HIPEC) have been used as local treatments in PC patients with gastric cancer, colorectal cancer, ovarian cancer, and mesothelioma accompanied by peritoneal pseudomyxoma (PMP) [6].

HIPEC involves the application of a concentrated chemotherapeutic solution at a high temperature into the peritoneal space of a patient with PC [7]. Compared to the IV route, this delivery route is less toxic, as only small quantities enter the systemic circulation. HIPEC is performed after CRS of tumors or lesions from the abdominal area [8]. Co-administration of hyperthermia with chemotherapeutic agents improves the cytotoxic effect of the drug [9]. A recent study reported an improvement in the cytotoxicity of mitomycin C when combined with hyperthermia [10]. HIPEC was prepared by mixing mitomycin C with 15 mg/m^2^ of body surface area in 4,500 mL of physiological saline. The mixed solution was circulated through a HIPEC pump for 90 minutes while maintaining 42 °C to 43 °C [2].

A 16-patient series reported an improved median survival time following aggressive CRS with HIPEC, ranging from 12 to 32 months. Furthermore, morbidity and mortality rates improved, from 14% to 55% and 0% to 19%, respectively [4]. The first use of the heat and chemotherapy combination used in the HIPEC technique can be traced back to Spratt et al. in 1980, who investigated its benefits for the treatment of PMP patients following total tumor removal [11,12]. In 2003, Sugarbaker described and developed the approach of CRS, followed by HIPEC [13]. In 2004, Sugarbaker’s technique appeared in the guidance published by the National Institute for Health Care Excellence (NICE) in the UK for the management of PMP [14].

The current study aims to describe the evidence-based published literature on the efficacy and safety of HIPEC following CRS in patients with PC originating from CRC. The review also aims to sum up the problems that can happen when HIPEC is used in these patients.

## Review

Protocol registration

The protocol was submitted for registration at PROSPERO, the International Prospective Register of Systematic Reviews, with application number #212449.

Search strategies

The current review was conducted in accordance with the Preferred Reporting Items for Systematic Reviews and Meta-Analysis (PRISMA) guidelines [15]. The literature search was performed across the PubMed, MEDLINE, EMBASE, clinicaltrials.gov, and Cochrane databases. No publication date restrictions were imposed; however, texts were restricted to those published in English. The search included all published articles, prospective studies, retrospective studies, and randomized controlled trials (RCTs) that utilized HIPEC with other systemic chemotherapeutic or surgical approaches for the treatment of CPC. Several keywords were used to conduct the database searches: "hyperthermic intraperitoneal chemotherapy," "HIPEC," "cytoreductive surgery," "colorectal cancer," "peritoneal carcinomatosis," and "colorectal peritoneal carcinomatosis."

Study selection and screening

All retrieved abstracts were screened independently by two teams of two researchers. The full texts that passed the initial screening process were further analyzed as per the inclusion criteria. Any disagreements in the selection or screening process were resolved by discussion and consensus with the supervisor and other researchers.

Inclusion and exclusion criteria

All published articles, prospective studies, retrospective studies, and RCTs that utilized HIPEC with other systemic chemotherapeutic or surgical approaches for the treatment of CPC were included.

Cancer treatment publications that did not involve HIPEC, as well as all reviews, editorials, and case reports, were excluded. Articles presented at conferences and articles that were not of the necessary quality were excluded.

Data synthesis and data extraction

A predefined Excel sheet (Microsoft Excel, Microsoft® Corp., Redmond, WA) was used to extract relevant data from the selected studies: (1) the general study characteristics (first author, year of publication, study design, and number of participants); (2) the main results, including survival outcomes for all patients; (3) the morbidity and mortality rates; and (4) the HIPEC technique used (method, drug, dosage, duration, and temperature).

Results

Study Selection

**Figure 1:**
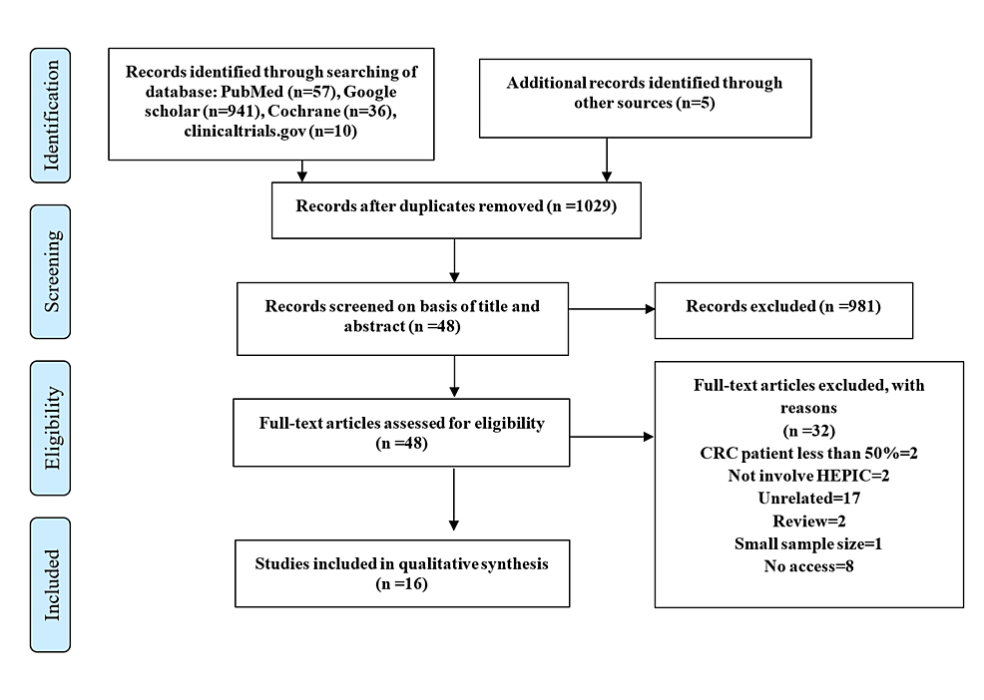
Prisma flowchart detailing article selection process.

Publications were retrieved from the PubMed (n = 57), Google Scholar (n = 941), Cochrane (n = 36), and clinicaltrials.gov (n = 10) databases, as well as from other sources (n = 5). Following the removal of duplicate studies, 1029 publications were identified. Following title and abstract screening, 48 publications were selected for full-text assessment, of which 16 were selected for inclusion in the current review’s qualitative and quantitative analyses (Figure 1). Of the 16 articles included in the review, four were RCTs, eight were retrospective studies, and four were retrospective and prospective studies.

Patient Characteristics

All studies addressed the treatment of CPC in patients with the primary PC type. Across the four RCTs, there were 287 and 285 patients in the HIPEC and non-HIPEC groups, respectively. The groups of patients included both sexes, and their ages ranged from 18 to 70 years. One of these studies was a follow-up to a previous study. In these studies, randomization was performed centrally by computer and stratified for presentation (primary or recurrent) and site (appendix, colon, or rectum) [16-19]. The eight retrospective studies involved the retrospective analysis of 746 patients: 700 patients had CPC originating from the colon and 46 from the rectum. Three studies were published only in abstract form. Both sexes were represented in the samples, and the patients’ ages ranged between 20 and 79 years [20-27]. The remaining four studies were retrospective and prospective analyses. Across these studies, a total of 312 patients were recruited into the HIPEC group. Both sexes were represented in the samples, and the patients’ ages ranged from 19 to 81 years [28-31]. The common chemotherapeutic regimen used across all studies was CRS followed by HIPEC using mitomycin (MMC ± cisplatin/doxorubicin) or oxaliplatin (LOHP) (LOHP ± irinotecan), with or without adjuvant systemic chemotherapy with fluorouracil-leucovorin.

Primary Outcomes

Overall survival: Thirteen studies reported overall survival rates, summarized in Table 1. Reported outcomes include overall survival, median overall survival, and survival of 1-5 years. Overall survival rates ranged from 63% to 93%; overall median survival ranged from 13 to 60.1 months; a one-year survival rate ranged from 70.7% to 96%; a two-year survival rate ranged from 28.3% to 100%; a three-year survival rate ranged from 20% to 74%; and a five-year survival rate ranged from 0% to 48.5% [16-31].

**Table 1:** Survival outcomes following HIPEC and CRS for colorectal peritoneal carcinomatosis patients. HIPEC: hyperthermic intraperitoneal chemotherapy, CRS: cytoreductive surgery.

Author	Year	Study type	No. of patients		Survival outcomes	
			HIPEC	Non-HIPEC	HIPEC	Non-HIPEC
Verwaal et al. [16]	2003	RCT	54	51	Median survival = 22.3 months	Median survival = 12.6 months
Verwaal et al. [17]	2008	RCT follow-up was updated in 2007	All Pt. still alive	All Pt. still alive	Median PFS = 12.6 months	Median PSF = 7.7 months
Quenet et al. [18]	2021	RCT	133	132	Median survival = 41.7 months 1-year survival = 86.9% 5-year survival = 39.4%	Median survival = 41.2 months 1-year survival = 88.3% 5-year survival = 36.7%
Klaver et al. [19]	2019	RCT	100	102	Overall survival = 93% Disease-free survival = 69%	Overall survival = 94.1% Disease-free survival = 69.1%
Elias et al. [20]	2006	Retrospective	30	-	Median survival = 60.1 months 3-year survival rate = 53% 5-year survival rate = 48.5%	-
Elias et al. [21]	2010	Retrospective	523	-	Median survival = 30.1 months 1-year survival rate = 81% 3-year survival rate = 41% 5-year overall survival = 27%	-
Baumgartner et al. [22]	2015	Retrospective	41	-	Median PFS = 9.69 months	-
Piso et al. [23]	2007	Retrospective	16	-	1-year OS rate = 96%	-
Park et al. [24]	2014	Retrospective	55	-	NR	-
de Boer et al. [25]	2019	Retrospective	27	-	Median survival = 22 months 1-year survival = 70.7% 2-year survival = 28.3%	-
Tonello et al. [26]	2019	Retrospective	36	-	Colonic group: Median survival = 47.83 month 3-year survival rate = 74% 5-year survival rate = 50%. Rectal group: Median survival = 22.0 months 3-year survival rate = 20% 5-year survival rate = 0%.	-
Kecmanovic et al. [27]	2005	Retrospective	18	-	Median survival time = 15	-
Yonemura et al. [28]	2013	Retrospective and prospective	87	-	Median survival = 24.4 months 5-year survival rate = 23.4%	-
Cavaliere et al. [29]	2006	Retrospective and prospective	120	-	Median survival = 19 month 2-year survival = 100% 3-year survival = 25.8 %	-
Pallas et al. [30]	2017	Retrospective and prospective	85	-	Median survival = 13 months 5-year survival rate = 23%	-
Bretchax et al. [31]	2010	Retrospective and prospective	20	-	Overall survival = 36%	-

In three of the four RCTs that used surgery only as the comparative group (one of the four was a follow-up study and, thus, was omitted), the HIPEC group demonstrated a statistically significant improvement in survival rate compared to the non-HIPEC group: 22.3 months and 12.6 months, respectively [16,17].

Three studies reported overall survival in patients with CRS separately. Overall median survival for this group of patients ranged from 12.6 to 41.2 months [16,18]. One study reported overall survival of 94.1%, while another reported a one-year survival rate of 88.3% and a five-year rate of 36.7% [18,19]. The overall median survival of patients in HIPEC groups was comparatively better than those in non-HIPEC groups.

Disease-free survival: Only one of the studies reported disease-free survival. Klaver et al. reported three-year disease-free survival in their HIPEC and non-HIPEC groups of 69% and 69.3%, respectively [19]. This difference was not significant.

Progression-free survival: Two studies reported median progression-free survival. Baumgartner et al. reported a median progression-free survival of 9.69 months [22]. Verwaal et al. reported median progression-free survival in their HIPEC and non-HIPEC groups of 12.6 and 7.7 months, respectively [17].

Mortality and Morbidity

Of the 16 studies, 12 reported morbidity rates (Table 2). The morbidity rate ranged from 4.4% to 65.4% [18-21,23-25,27-31]. Reported toxicity and complications are summarized in Table 3. Hematological toxicity (e.g., leukopenia and anemia) and gastrointestinal fistulas were the most-reported complications. Eleven papers reported mortality rates that ranged from 0% to 13% [16,17,20,21,23-25,27-30]; grade complications according to the National Cancer Institute's Common Toxicity Criteria [32].

**Table 2:** Mortality and morbidity rates after HIPEC and CRS for peritoneal carcinomatosis from colorectal cancer. HIPEC: hyperthermic intraperitoneal chemotherapy, CRC: cytoreductive surgery.

Author	Year	No. of patients	Mortality rate, n (%)	Morbidity rate, n (%)
Verwaal et al. [16]	2003	54	8	NR
Verwaal et al. [17]	2008	All Pt. still alive	NR	NR
Quenet et al. [18]	2021	133	2.6	65.4
Klaver et al. [19]	2019	87	NR	14
Elias et al. [20]	2006	30	0	40
Elias et al. [21]	2010	523	3	31
Baumgartner et al. [22]	2015	41	NR	NR
Piso et al. [23]	2007	16	0	34
Park et al. [24]	2014	55	13	47
de Boer et al. [25]	2019	27	0	48.2
Tonello et al. [26]	2019	36	NR	NR
Kecmanovic et al. [27]	2005	18	0	4.4
Yonemura et al. [28]	2013	87	0.7	42.9
Cavaliere et al. [29]	2006	120	3.3	22.5
Pallas et al. [30]	2017	85	2	43
Bretcha-Boix et al. [31]	2010	20	NR	40

**Table 3:** List of major toxicities and complications in patients with colorectal peritoneal carcinomatosis treated with CRS and HIPEC. HIPEC: hyperthermic intraperitoneal chemotherapy, CRC: cytoreductive surgery. Grade 1 - Mild; asymptomatic or mild symptoms; clinical or diagnostic observations only; intervention not indicated. Grade 2 - Moderate; minimal, local or noninvasive intervention indicated. Grade 3 - Severe or medically significant but not immediately life-threatening; hospitalization or prolongation of hospitalization indicated. Grade 4 - Life-threatening consequences; urgent intervention indicated.

	n % (Grade I to IV)
Fever	6 (grade 3)
Leukopenia	2–15 (grade 3–4)
Thrombocytopenia	4 (grade 3)
Anemia	11 (grade 3)
Neuropathy	4 (grade 3)
Pleural effusion	2 (grade 3)
Pulmonary embolus	4 (grade 4)
Pneumonia	2–6 (grade 3)
Renal obstruction	4 (grade 3)
Anuria (acute tubular necrosis)	6 (grade 4)
Cardiac arrhythmia	2 (grade 4)
Heart failure	4–8 (grade 3–4)
Hemorrhage	6–10 (grade 2–4)
GI fistula	2–15 (grade 2–4)
Pancreatitis	2 (grade 3)
Catheter infections	6–16 (grade 3)
Abscesses	4–5.3 (grade 3-4)
Peritonitis	3 (grade 3)
Sepsis	10 (grade 2–4)
Psychological disorders	4–6 (grade 3–4)

HIPEC Technique

The heterogeneity of the trials included in this review must be taken into account when interpreting their results. This includes the HIPEC method, chemotherapy, regimen, and temperature used. All variations are summarized in Table 4.

**Table 4:** Variations in hyperthermic intraperitoneal chemotherapy technique.

Author	Year	Method	Drug	Dosage	Duration	Temp
Verwaal et al. [16]	2003	Open	MMC	17.5 mg/m^2^ followed by 8.8 mg/m^2^ every 30 minutes. The total dose was limited to a 70 mg maximum	90 minutes	40 °C
Verwaal et al. [17]	2008	Open	MMC	17.5 mg/m^2^ followed by 8.8 mg/m^2^ every 30 minutes. The total dose was limited to a 70 mg maximum.	90 minutes	41–42 °C
Quenet et al. [18]	2021	Closed	LOHP	360 mg/m^2^	30 minutes	NR
Klaver et al. [19]	2019	Open or closed	LOHP	460 mg/m^2^	30 minutes	42–43 °C
Elias et al. [20]	2006	Open	LOHP	460 mg/m^2^	30 minutes	43 °C
Elias et al. [21]	2010	Open or closed	MTC + cisplatin OR LOHP + irinotecan	MTC 30–50 mg/m^2^ + cisplatin 50–100 mg/m^2^ OR LOHP 360–460 mg/m^2^ + irinotecan 200 mg/m^2^	30–90 minutes	41–43 °C
Baumgartner et al. [22]	2015	Closed	MTC	MTC 10 mg/L	90 minutes	41 °C
Piso et al. [23]	2007	Open	MTC + doxorubicin	MTC 20 mg/m^2^ with doxorubicin 15 mg/m^2^	NR	41–42 °C
Park et al. [24]	2014	Closed	MMC	NR	90 minutes	42–43 °C
Kecmanovic et al. [27]	2005	Open	MTC	MTC (12.5 mg/m^2^, maximum. 25 mg for males; 10.0 mg/m^2^, maximum. 20 mg for females)	NR	43.8 °C
Yonemura et al. [28]	2013	NR	MTC + cisplatin	MTC 20 mg/body weight and cisplatin 100 mg/body weight	60 minutes	42–43 °C
Cavaliere et al. [29]	2006	Open or closed	MTC + cisplatin	MTC (3.3 mg/m^2^/L) and cisplatin (25 mg/m^2^/L)	60–90 minutes	41.5–43 °C
Pallas et al. [30]	2017	Open or closed	MTC	20 mg/m^2^	90 minutes	42.5–43 °C
Bretcha-Boix et al. [31]	2010	Closed	MTC or LOHP	MTC 10–12.5 mg/m^2^ or LOHP 360 mg/m^2^	40–90 minutes	42 °C

Prognostic Factors

Seven out of sixteen studies did an analysis of the factors that may affect survival. The two most frequently discovered factors were the peritoneal carcinomatosis index (PCI) score and the degree of completeness of cytoreduction (CC). PCI was found to be an important prognostic factor in four studies. Elias et al. reported that PCI > 15 was the threshold level for a significantly poor prognosis. The survival of patients with PCI ≤ 10 was significantly better than that of patients with PCI ≥ 11, as reported by Yonemura. Pallas et al. reported that patients with PCI of more than 20 had no chance to survive more than five years. Kecmanovic et al. reported that the PCI was equal to or less than 13 in 10 patients, and their median survival time was 16.8 months, statistically significantly lower than the median survival time of 6.9 months for the eight patients with PCI greater than 13 [19,26,27,29]. The completeness of cytoreduction was found to be a prognostic factor in six studies. The five-year overall survival rates recently reported by expert centers after CCR-0: 43% for the 59 patients reported by Verwaal et al., 45% in Verwaal et al., 48% for the 30 patients reported by Elias et al., 30% in Elias et al., 43% for the 51 patients reported by Pallas et al., and 20% in Yonemura et al. [15,16,19,20,27,29].

Discussion

Over the last century, aggressive peritoneal treatments, such as HIPEC and CRS, have been implemented in an attempt to enhance survival and quality of life for patients with manifestations of intra-abdominal malignancies. These procedures assume that cancer that is isolated to the peritoneal cavity is a loco-regional disease. CRS is a complicated surgical procedure that involves a peritonectomy and the resection of involved viscera, with the intention of leaving only microscopic amounts of disease [13]. The biological rationale for intraperitoneal delivery is based on research that has demonstrated the pharmacokinetic benefits of this delivery method. The peritoneal-plasma barrier allows for a high concentration gradient of chemotherapeutic drugs between the peritoneal cavity and the systemic circulation and blood drainage from the peritoneal cavity via the portal system. This provides a "first-pass" effect through the liver, reducing systemic toxicity while simultaneously increasing intrahepatic concentrations. Experimental evidence suggests that malignant cells are more vulnerable to the effects of hyperthermia in the range of 41-43 °C, resulting in accelerated cell death. Furthermore, evidence suggests a correlation exists between heat and increased cytotoxicity of some chemotherapeutics used during HIPEC [33].

Possible advantages of using HIPEC compared to intravenous chemotherapy include increased exposure of chemotherapeutic drugs to peritoneal malignant cells, increased drug penetration into tissues, synergistic effects of chemotherapy, and independent cytotoxic effects of hyperthermia [34]. CRS and HIPEC may increase the risk of surgical complications and toxicity, which may lead to postoperative bleeding, anastomosis leaks, bowel perforations, wound dehiscence, etc. HIPEC may lead to nephrotoxicity or hepatotoxicity [35].

CRS/HIPEC was recommended in selected patients as a curative-intent treatment option for CRC: (i) Complete or near-complete cytoreduction (CC0-1) residual individual tumor areas no bigger than 2.5 mm in diameter should be achievable. (ii) The presence of distant metastases other than CRC (lung and liver) is a contraindication for CRS/HIPEC. (3) Older age might be a relative contraindication for treatment with CRS/HIPEC [17,36,37].

Four RCTs [16-19], eight retrospective studies [20-27], and four retrospective and prospective studies [28-31] were found and selected during the literature screening process. In the study by Verwaal et al. and Verwaal et al., randomization was performed centrally by computer and stratified for the presentation site (colon or rectum) [16,17]. The common chemotherapeutic regimen used across the studies was CRS followed by HIPEC using mitomycin (MMC ± cisplatin/doxorubicin) or oxaliplatin (oxaliplatin ± irinotecan), with or without adjuvant systemic chemotherapy using fluorouracil-leucovorin. Patients treated with a combination of HIPEC and CRS demonstrated significantly better survival rates than those who received CRS alone. Furthermore, the overall median survival of patients treated with the combined HIPEC and CRS approach was comparatively better than those who received CRS alone. There was insufficient evidence comparing the two groups to ascertain whether this treatment regimen was associated with a higher or lower morbidity rate. However, the mortality associated with this treatment regimen appeared to be low. Nevertheless, further studies are needed to validate these results. The conclusions of these trials differed, but there were significant methodological differences. The control group in some experiments did not receive CRS, while the comparator was always a mixture of HIPEC and CRS. Therefore, it is unclear whether CRS, HIPEC, or the combination of the two is responsible for the survival advantage conferred to patients who received the combined regimen. Despite this uncertainty, the American Society of Peritoneal Surface Malignancies recommends that HIPEC in colorectal cancer patients be standardized using MMC at a dosage of 40 mg and a temperature of 42 °C for a total of 90 minutes of perfusion [38].

## Conclusions

CPC treatments using HIPEC and CRS were developed to increase patient survival and quality of life. In the present study, the overall median survival of patients treated with a combined HIPEC and CRS regimen was comparatively better than those who received CRS alone. There was no significant difference in overall disease-free survival, whereas progression-free survival was shown to be higher in patients treated with the combined HIPEC and CRS regimen. However, further RCTs with adequate patient numbers, comparable treatment concepts, and similar inclusion criteria-controlled trials are required to validate these results.
